# Antioxidant Activities of Aqueous Extracts and Protein Hydrolysates from Marine Worm Hechong (*Tylorrhynchus heterochaeta*)

**DOI:** 10.3390/foods11131837

**Published:** 2022-06-22

**Authors:** Wenxia Zhang, Zexiong Wang, Kumar Ganesan, Yingzhi Yuan, Baojun Xu

**Affiliations:** 1 Programme of Food Science and Technology, BNU-HKBU United International College, Zhuhai 519087, China; zhangwenxia0118@gmail.com (W.Z.); zw192@georgetown.edu (Z.W.); kumarg@hku.hk (K.G.); 2 School of Chinese Medicine, Li Ka Shing (LKS) Faculty of Medicine, University of Hong Kong, Sassoon Road, Hong Kong 999077, China; 3 Department of Biochemistry, University College London, London WC1E 6BT, UK; zcbtyy3@ucl.ac.uk

**Keywords:** hechong, glycoprotein, protein hydrolysates, antioxidants

## Abstract

Hechong (*Tylorrhynchus heterochaeta*) is an edible marine worm widely distributed in the estuary area. The objective of this study is to determine the antioxidant activities of extracts and protein hydrolysates from Hechong. Results showed that the aqueous extracts of steamed Hechong had the highest antioxidant values using the methods of DPPH, ABTS, and FRAP testing (76.29 μmol TE/g, 181.04 μmol TE/g, and 10.40 mmol Fe^2+^/100 g, respectively). Furthermore, protein hydrolysates of Hechong were observed significant antioxidant activities when compared to crude Hechong. The purification was carried out by DEAE-52 cellulose and Sephadex G-100 column chromatography. The microspatial structure of glycoprotein showed fibrous shapes and cracks with uniform distribution. The study has concluded that the extract and protein hydrolysates of Hechong have significant antioxidant activities, which is merited to be further investigated in the food and pharmaceutical fields.

## 1. Introduction

Hechong (*Tylorrhynchus heterochaeta*) is a marine worm belonging to the phylum Annelida, the class of Polychaete, and the family of Nereididae, and are typically habitat in the sandy soil where salty water and freshwater meet or paddy field in the estuary [1]. They are widely found in the coastal regions of Guangdong, Guangxi, Zhejiang, and Fujian Provinces in China, also the provinces in the northern coastal plains of Vietnam. In the local market, Hechong is considered a high nutritional value food and is a popular traditional food in Pearl River Delta because of its nutrition and delicious taste [2]. Hechong is rich in proteins, essential amino acids, unsaturated fatty acids, vitamin B, and trace elements including, calcium, iron, and selenium [3]. The contents of unsaturated aliphatic acids such as eicosapentaenoic acid, arachidonic acid, and linoleic acid in Hechong are higher in quantities when compared to the other Nereididae, *Perinereis aibuhitensis*, and *Neanthes japonica* [4]. In recent years, the active substances obtained from other Nereididae have been well documented, which possess antioxidant, anti-tumor, anti-fatigue, and anti-thrombotic functions [5,6]; however, few researchers have been studied Hechong and its active extracts [1,7].

At present, the basic investigations on Hechong are very limited. Earlier studies have been established on Hechong and its nutrient composition [3], a histological structure, [8], amino acid sequence and polypeptide chain composition of hemoglobin [9,10], and environmental quality indicators [2]. Earlier, the functional properties of other species in Nereididae and their extracts or hydrolysates have been well documented [11]. For instance, two sulfated polysaccharides were isolated from *Perinereis aibuhitensis* through the digestion of trypsin and papain followed by the fractionation and purification via anion-exchange and gel-permeation chromatography. The purified polysaccharides exhibited remarkable anticoagulant activities in vitro and antithrombotic effects in vivo [12]. The identified peptide was Pro-Val-Glu-Arg-Lys which prevented the formation of carrageenan-induced tail thrombosis in mice, FeCl3-induced arterial thrombosis in rats, and ADP-induced platelet aggregation [11]. The active components extracted from *Nereis virens* showed positive activity in inhibiting the growth of mouse melanoma A375 cells. Previously, the extracts were extracted with ethyl acetate and separated by silica gel column via segmented gradient elution. One of the fractions that were eluted with petroleum ether/ethyl acetate (3:7, *v*/*v*) showed a noteworthy antitumor activity [13].

In recent years, commercial interests in functional food and its ingredients have developed progressively because of the improved consumer awareness of the relationship between healthy eating, nutrition, and lifestyle [14]. People are seeking many ways to achieve a better diet nowadays. Hechong has also been highlighted in ‘Traditional Chinese Medicine’ for healing several illnesses and is used in foods like salty, cold, sweet, and aphrodisiac tonic [1]. They are used in primary medicine for beriberi and renal problem, enhance milk secretion for lactating mothers, decrease neurasthenia and spleen embolism in children, reduce the menace of atherosclerosis, and improve prostate functions in adult males [1].

Enzymatic hydrolysis is the most common method to degrade the complex proteins into several small peptides and short amino acid chains [15]. The protein hydrolysates have distinct biological activities, including antioxidant, anticancer, antimicrobial, and antidiabetic activities [16]. Thus, the study of enzymatic hydrolysates is highly advantageous and required in the research field, especially in the functional food of Hechong. Protein hydrolysate generally has diverse functional activities based on the presence of molecular size, amino acid composition, and amino acid sequences [17].

Glycoprotein is a covalent compound formed by covalent bonds between proteins and oligosaccharides [18,19]. In nature, the glycoprotein is transmembrane proteins widely present in the kingdoms of animals, plants, and microorganisms, and are important substances for all living activities. Glycoprotein has diverse functions of regulating immunity, antitumor, anti-inflammation, hypoglycemic, hypolipidemic, antioxidant, anti-aging, and other biological activities [19]. Hence, the investigations on exploring novel functional and nutritional values of Hechong are highly indispensable.

Therefore, the objective of the present study was aimed to obtain different extracts by hot water, 70% acetone extraction, and hydrolysates by proteases such as alcalase, trypsin, papain, neutrase, and pepsin. The extracts and protein hydrolysate of Hetchong were evaluated for antioxidant assay by DPPH, ABTS, and FRAP. Furthermore, purity testing, space structure observation, and molecular weight measurement of glycoproteins were achieved.

## 2. Materials and Methods

### 2.1. Mateirals

The sample of fresh Hechong (*Tylorrhynchus heterochaeta*) was shown in Supplemental Figure S1A. In this study, one sample was provided by Estuarine Fisheries Research Institute in Zhuhai, Guangdong Province, China, and the other sample was harvested from Vietnam.

### 2.2. Chemicals and Reagents

The water used in this study was deionized (18 MΩ·cm) from Water Purification System. Sephadex G-100, DEAE-52, 2,2-diphenyl-1-picrylhydrazyl, 2′-azino-bis (3-ethylbenzothiazoline-6-sulfonic acid, 2,4,6-tri(2-pyridyl)-s-triazine, 6-hydroxy-2,5,7,8-tetramethlchroman-2-carboxylic acid, casein, glacial acetic acid, Tris-base, alcalase, trypsin, papain, neutrase, pepsin, and ammonium persulfate were purchased from Yuanye Biotech Co. Shanghai, China. Absolute ethanol (analytical grade), acetic acid, acetone, ferric chloride, ferrous sulphate, glucose, methanol (analytical grade), perchloric acid, phenol (redistilled reagent grade), potassium per-sulphate, potassium sulphate, sodium acetate, and sodium hydroxide were purchased from DaMao Chemical Co. Tianjin, China. Boric acid, copper sulfate, hydrochloric acid, methylene blue indicator, methyl red indicator, petroleum ether, sulfuric acid, and sodium thiosulfate were purchased from Guangzhou Chemical Co. (Guangzhou, China). Bovine serum albumin, Tris-HCl buffer, SDS, tetramethylethylenediamine were obtained from Sigma Chemical Co. (St. Louis, MO, USA) RIPA lysis buffer, acrylamide/bis-acrylamide mix, ladder protein, were purchased from Thermo Fisher Scientific Co. (Shanghai, China). All other chemicals were of analytical grade.

### 2.3. Preparation of Different Extracts of Hechong

Several treatments were combined and obtain six groups of Hechong extracts from raw material based on the method of Chen et al. [20] with modifications. As shown in Supplemental Table S1, Group 1- raw Hechong freeze-drying powder was prepared by freeze-drying raw material directly via a vacuum freeze drier (FreeZone Benchtop, Labconco Company, Kansas, MO, USA). Group 2 was obtained by extracting Hechong freeze-dried powder using 70% acetone. Briefly, added 0.1 g accurately weighed ground dry sample powder with 1 mL of acetone/water/acetic acid (70:29.5:0.5, *v*/*v*/*v*) solvent (70% acetone). The sample was extracted twice via shaking in an orbital shaker for 3 h and another 12 h by setting in the dark overnight. Centrifuged at 6000 rpm for 10 min (Cence Co., Suzhou, China), combined both extracts, and recorded as final volume (FV) of the extracts. Group 3 was hot water extracts of Hechong- Briefly, Hechong was extracted using a water bath at 90–100 °C for 2 h and added distilled water at the ratio of 1:5 of solid to liquid. Then the extracts were filtered and centrifuged; the supernatant was taken and maintained at −80 °C refrigerator and obtained a freeze-dried powder. Group 4 was obtained by extracting Hechong aqueous extracts from freeze-dried powder using 70% acetone, which was followed as similar to Group 2. Group 5 was aqueous extracts of steamed Hechong- The raw material was steamed for 15 min followed by the extraction using hot water similar to Group 3. Group 6 was obtained by extracting steamed Hechong aqueous extracts from freeze-dried powder using 70% acetone, which also followed the steps as like as Group 2. All powder samples were stored at −80 °C and all 70% acetone extracts were stored at 4 °C until further use.

### 2.4. Preparation of Protein Hydrolysates

The Hechong enzymatic hydrolysates were prepared based on the methods of Altinelataman [21] and Tan [22] with modifications. 100 g of raw Hechong were minced with distilled water (1:1, *w/v*) by a Midea home type blender. The protein contents of the homogenate of Hetchong obtained from China and Vietnam were determined by Kjedahl [23], which were 5.75% and 5.64% respectively. The homogenate was taken into an Erlenmeyer flask and heated at 85–90 °C for 15 min. After cooling down, a 0.1 M sodium phosphate buffer solution containing different enzymes was added at a ratio of 1:1.5 of solid to liquid. The buffer solution usually provided the optimum pH to all enzymes, and the enzymes were added to the buffer solution to initiate the hydrolysis reaction at the enzyme-to-substrate ratio (E:S) of 1:50 (*w/w*). The substrate was the protein of Hechong homogenate. Different enzymatic processes were carried out in the water bath shaker using different optimum temperatures: 55 °C at pH 8.0 for alcalase and trypsin, 50 °C at pH 6.0 for papain, 50 °C at pH 7.0 for neutrase, and 37 °C at pH 2.0 for pepsin. Enzymatic hydrolysates were sampled at the time of 30 min, 60 min, 120 min, and 180 min, and the hydrolysis was terminated by heating at 90 °C for 5 min. For each group, the hydrolysates in 0 min (without enzyme addition) were collected as a control. After cooled down, the hydrolysates were centrifuged at 7000 rpm, at 4 °C for 15 min. The supernatants were frozen at −80 °C refrigerator and then freeze-dried to obtain a powder. All powder samples were stored at −80°C for experimental use.

### 2.5. Determination of DPPH Free Radical Scavenging Activity

The ability of Hechong extracts and hydrolysates to scavenge free radicals 2,2-diphenyl-1-picrylhydrazyl (DPPH) was measured according to the method employed by Luo et al. [24] with slight modifications. The value of DPPH was presented as Trolox equivalents (μmol TE/g sample powder) with the calibration curve of Trolox with a linearity range from 50 μg/mL to 750 μg/mL (*R^2^* > 0.999).

### 2.6. Determination of ABTS Free Radical Scavenging Activity

The ability of Hechong extracts and hydrolysates to scavenge free radicals 2′-azino-bis (3-ethylbenzothiazoline-6-sulfonic acid) (ABTS) was measured according to the method of Ismail et al. [25] with slight modifications. The value of ABTS was presented as Trolox equivalents (μmol TE/g sample powder) with the calibration curve of Trolox with a linearity range from 50 μg/mL to 1000 μg/mL (*R^2^* > 0.999).

### 2.7. Determination of Ferric Ion Reducing Antioxidant Power

The determination of ferric ion reducing antioxidant power (FRAP) was followed according to the earlier work performed by Luo et al. [24] with slight modifications. FRAP value was presented as mmoles of Fe^2+^ equivalents per 100 g of sample (mmol FE/100 g sample powder) with the calibration curve of Fe^2+^ with a linearity range from 0.1 mM to 1.0 mM (*R^2^* > 0.9999).

### 2.8. Determination of Molecular Weight Distribution

The molecular weight distribution of Hechong protein hydrolysates in both the China and Vietnam group was determined using the SDS-PAGE method of Chen [26], Firmansyah, and Abduh [16] with modifications. Polyacrylamide gel for electrophoresis was prepared by mixing a solution of acrylamide/bis-acrylamide (30%/8–0.8%, *w/v*), distilled water, Tris-HCl buffer (0.5 M, pH 6.8, staking gel) with Tris-HCl buffer (1.5 M, pH 8.0, separating gel), SDS, ammonium persulfate (10%, *w/v*) and tetramethylethylenediamine. 30 mg freeze-dried powder of protein hydrolysates were weighed and dissolved in 500 μL RIPA lysis buffer. After centrifuging at 10,000 rpm for 20 min, 80 μL of supernatant was added to a 20 μL loading buffer. The sample was incubated in a boiling water bath for 5 min. Electrophoresis was operated using an SDS-PAGE device (PowerPac Basic, Bio-Rad Co., Hercules, CA, USA) at a voltage of 120 V for 90 min at 25 °C. A protein marker of 4.6–180 kDa was used to determine the molecular weight distribution for comparison. The gel coloring process was carried out for 12 min, and the colored gel was rinsed and socked in a de-staining solution consisting of methanol (10% *v/v*), glacial acetic acid (10% *v/v*), and distilled water (80% *v/v*) for another 12 h until the bands showed clearly.

### 2.9. Extraction and Purification of Glycoprotein from Hechong

The glycoprotein of Hechong was extracted and purified by hot water extraction, DEAE-52 cellulose column chromatography, and Sephadex G-100 column chromatography. The fresh Hechong samples were homogenized and washed. About 50 g of Hechong samples were boiled in 500 mL of boiling water for 60 min. The filtrate was then freeze-dried using a freeze dryer (Labconco Corporation, Kansas, MO, USA), weighed, and stored in the refrigerator at 4 °C. The crude protein powder was labeled as HGP-1, which is shown in supplemental Figure S1B.

Accurately 0.3 g of HGP-1 samples were weighed and dissolved with 10 mL of water, followed by centrifugation (Cence Co., Suzhou, China), and obtained the supernatant. The purification was achieved by DEAE-52 cellulose column chromatography according to the earlier publication [27]. The eluent solution was sodium bicarbonate (0.1 mol/L) with an eluant velocity of 1.0 mL/min, and the fractions were collected into the tube every 2 min. The carbohydrate part was detected at 490 nm by the phenol-sulfuric acid method. 0.1 mL of phenol (6%) was added into each tube containing 0.2 mL samples and was kept in a water bath. About 0.5 mL of H_2_SO_4_ was added into a mixture and mixed on the Vortex test tube mixer and kept at room temperature for 30 min. The absorbance was measured at 490 nm by a UV-visible light spectrophotometer (JingKe Instrument Co., Shanghai, China). Then the protein was determined at 280 nm by the UV-visible light spectrophotometer. For the elution curve, the collected tube number (T) was on *X*-axis, and absorbance (A) was on *Y*-axis. The coincident absorption peak of the two curves was collected and freeze-dried to gain crude glycoprotein HGP-2, which is shown in supplemental Figure S1C.

The freeze-dried powder HGP-2 was further isolated and purified by Sephadex G-100 column chromatography. The collected glycoprotein HGP-2 was dissolved into 1 mL of water with a 1 mL loading quantity. The eluent was pure water with an eluant velocity of 1.0 mL/min, and the fractions were collected every 2 min into a tube. After making the elution curve, the coincident absorption peak of the two curves was collected and freeze-dried to gain glycoprotein HGP-3, which is shown in supplemental Figure S1D.

### 2.10. Morphology Measurement of Glycoprotein from HECHONG

After freeze-drying, the surface morphology measurement of purified HGP-3 was observed by the field emission scanning electron microscope (SEM, Carl Zeiss, Jena, Germany) with an accelerating voltage. Furthermore, the measurement of the three-dimensional morphology of HGP-3 was examined by atomic force microscopy (AFM, Bruker Dimension Icon, Santa Barbara, U.S.A.). Both HGP-1 and HGP-3 were tested by the method of SDS-PAGE according to the previous publication [26]. The separation gel concentration was 12 mol/L with an electrode buffer of 1.5 mol/L Tris-Gly-SDS solutions at pH 8.3. The loading quantity was 7 µL, and the initial voltage was 80 V and then electrophoresis was conducted for 2 h at 120 V after separation gel adding. The stain was Coomassie brilliant blue (CBB) R-250 and the de-staining solution was 7% glacial acid. The molecular weight of the glycoprotein was calculated using its relative electrophoretic mobility.

### 2.11. Statistical Analysis

Analyses were performed in triplicates, and results were expressed as means ± standard deviations on a dry weight basis. For multiple group comparisons, a one-way analysis of variance (ANOVA) was conducted by Duncan’s post hoc test and applied for determining the significance (*p* < 0.05) differences. Statistical analysis was carried out using Microsoft 2016 package and SPSS software (version 25.0, SPSS Inc., Chicago, IL, USA).

## 3. Results

### 3.1. DPPH Free Radical Scavenging Capacity of the Extracts and Protein Hydrolysates from Hechong Mateirals

**Figure 1:**
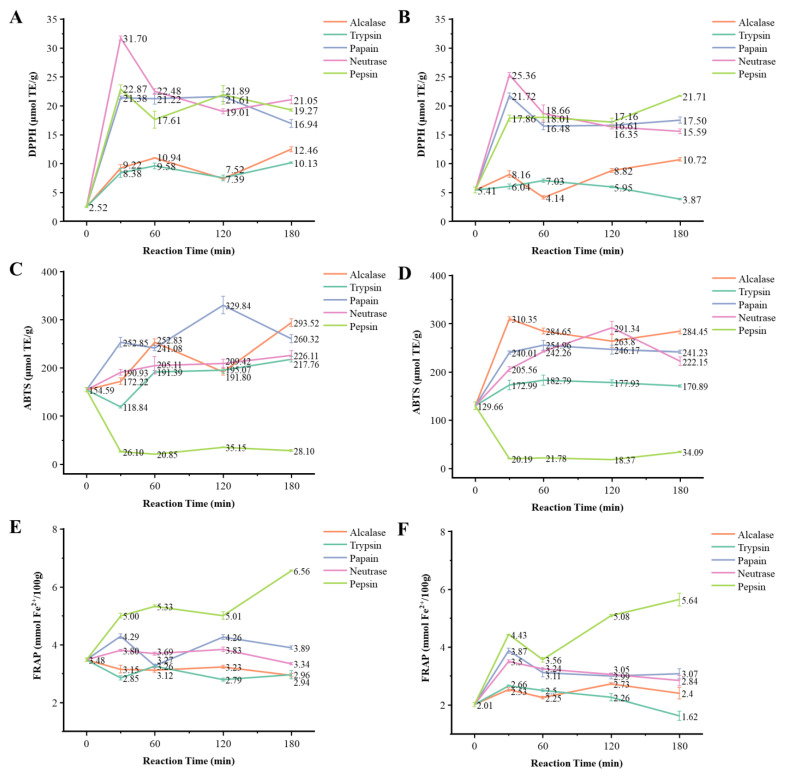
Free radical scavenging capacities of DPPH, ABTS, and FRAP of different Hechong hydrolysates in China and Vietnam group concerning enzymatic hydrolysis time (0 min, 30 min, 60 min, 120 min, and 180 min). The changes in DPPH value, ABTS value, and FRAP value of the China group illustrated in (**A**,**C**,**E**). The changes in DPPH value, ABTS value, and FRAP value of the Vietnam group illustrated in (**B**,**D**,**F**). Data were expressed as Mean ± S.D. (*n* = 3) on a dry weight basis.

DPPH free radical scavenging activity of extracts and hydrolysates from Hechong grown in China and Vietnam was significantly (*p* < 0.05) different (Table 1). In both China and Vietnam group, the steamed aqueous extracts had the highest DPPH value (64.15 μmol TE/g and 76.29 μmol TE/g, respectively), followed by raw aqueous extracts (52.07 μmol TE/g and 60.68 μmol TE/g, respectively). Similarly, the extracts with the lowest DPPH values occurred in 70% acetone extracts of raw freeze-dried powder (4.21 μmol TE/g and 2.62 μmol TE/g, respectively). For the Hechong hydrolysates, the neutrase hydrolysates obtained in 30 min had the strongest DPPH scavenging activity in both China and Vietnam group (31.70 μmol TE/g and 25.36 μmol TE/g, respectively), which were significantly (*p* < 0.05) different from the control sample without enzyme addition (2.52 μmol TE/g and 5.41 μmol TE/g, respectively) (Figure 1). Overall, the hydrolysates obtained from papain, neutrase, and pepsin treatments performed better DPPH scavenging activity than the hydrolysates obtained from alcalase and trypsin treatment.

### 3.2. ABTS Free Radical Scavenging Capacity of the Extracts and Protein Hydrolysates from Hechong

ABTS free radical scavenging activity of extracts and hydrolysates from Hechong grown in China and Vietnam was significantly (*p* < 0.05) different (Table 1). ABTS scavenging activity was performed almost the same in both China and Vietnam groups. The steamed aqueous extracts had the strongest ABTS scavenging activity (184.75 μmol TE/g and 181.04 μmol TE/g, respectively). Similarly, the raw aqueous extracts were also performed well with the values of 114.39 μmol TE/g and 135.00 μmol TE/g, respectively, followed by 70% acetone extracts of steamed Hechong (61.76 μmol TE/g and 71.01 μmol TE/g, respectively), 70% acetone extracts of raw Hechong (54.00 μmol TE/g and 58.25 μmol TE/g, respectively), and the raw Hechong freeze-dried powder (43.53 μmol TE/g and 53.24 μmol TE/g, respectively). The extracts with the lowest ABTS values were found in 70% acetone extracts of raw freeze-dried powder (11.04 μmol TE/g and 6.14 μmol TE/g, respectively). For the Hechong hydrolysates in the China group, the papain hydrolysates were obtained in 120 min, which had the strongest ABTS scavenging activity (329.84 μmol TE/g). Likewise in the Vietnam group, the strongest one was alcalase hydrolysates obtained in 30 min (310.35 μmol TE/g) (Figure 1). Overall, the hydrolysates obtained from alcalase, trypsin, papain, and neutrase treatment had a significant ABTS value (*p* < 0.05) when compared to the control group. However, the pepsin hydrolysates had a significantly reduced ABTS value (*p* < 0.05) when compared to the control.

### 3.3. FRAP Values of the Extracts and Protein Hydrolysates from Hechong

The FRAP values of extracts and hydrolysates from Hechong grown in China and Vietnam were significantly (*p* < 0.05) different. In the China group, raw and steamed aqueous extracts had the largest FRAP value of 6.77 mmol Fe^2+^/100 g and 10.40 mmol Fe^2+^/100 g respectively (Table 1). 70% Acetone extracts of raw freeze-dried powder had the lowest value of 1.18 mmol Fe^2+^/100 g and 0.64 mmol Fe^2+^/100 g, respectively. For the Hechong hydrolysates, the pepsin hydrolysates obtained in 180 min had the highest FRAP value in both China and Vietnam group (6.56 mmol Fe^2+^/100 g and 5.64 mmol Fe^2+^/100 g, respectively) (Figure 1). In the Vietnam group, all the hydrolysates had significantly higher FRAP values (*p* < 0.05) when compared to the control group, however, the trypsin hydrolyzates were obtained at 180 min (1.62 mmol Fe^2+^/100 g). Similarly, in the China group, the FRAP value of the control group was in the middle position (3.48 mmol Fe^2+^/100 g), half of the hydrolysates had higher FRAP values, and the remaining samples had minimum significant (*p* < 0.05). Overall, in both China and Vietnam group, pepsin hydrolysates had significantly (*p* < 0.05) better performance in FRAP.

### 3.4. Characterization of Protein Hydrolysates from Hechong Grown in China

**Figure 2:**
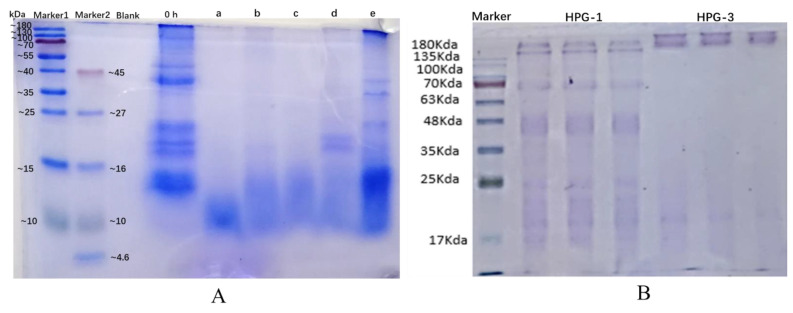
(**A**) Molecular weight determination of Hechong sample after enzymatic hydrolysis: without enzyme (0 h), and hydrolysates obtained in reaction time of 180 min with treatments of different enzymes: (a) alcalase, (b) trypsin, (c) papain, (d), neutrase, (e) pepsin. (**B**) The protein electrophoretogram of HGP-1 and HGP-3.

The molecular weight determination of Hechong was evaluated using the hydrolysates of Hechong by the method of SDS-PAGE. The results were shown in Figure 2. Overall, protein bands in the control sample (0 h) appeared in molecular size ranged about 14–180 kDa. The hydrolysates of Hechong showed an obvious reduction in molecular weight distribution. In particular, the molecular weight of Hechong hydrolysates treated by (a) alcalase, (b) trypsin, and (c) papain were the concentration ranges of about 10–16 kDa. For (d) neutrase treatment, two more protein bands were appearing in the range of 16–27 kDa, while the molecular weight of the (e) pepsin group was distributed in the range of 10–45 kDa. The protein electrophoretogram of HGP-1 and HGP-3 was exhibited in Figure 2B. From left to right, triplicate groups of HGP-1, and HGP-3 markers moved the runway. In the runway, seven staining bands in the HGP-1 and only a single band in the HGP-3.

### 3.5. Extraction and Purification of Glycoprotein from Hechong

**Figure 3:**
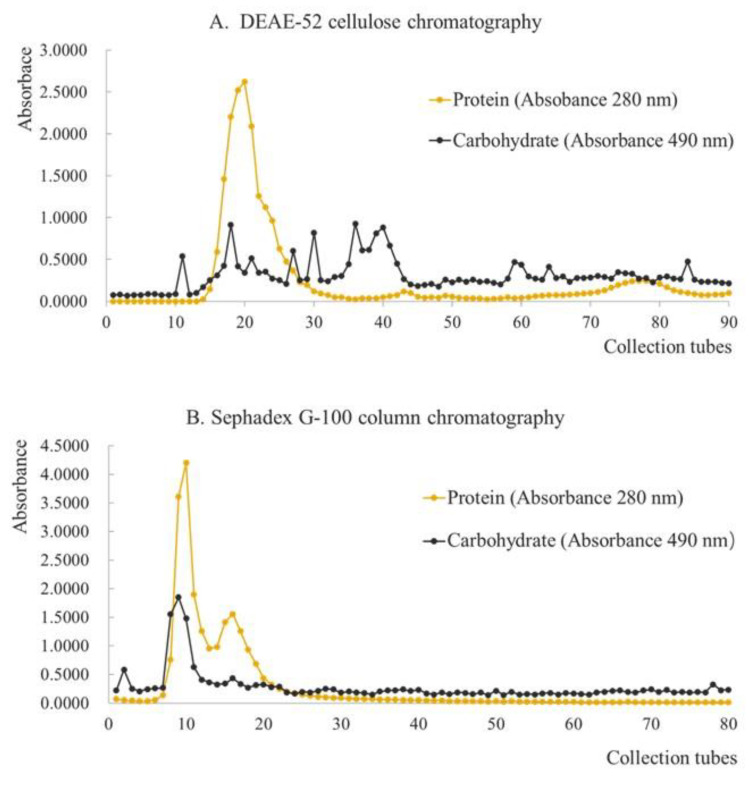
The result of DEAE-52 cellulose column chromatography of HGP-1 (**A**) Sephadex G-100 column chromatography of HGP-2 (**B**).

The crude protein HGP-1 of Hechong yield 4.124% glycoproteins. The results of DEAE-52 cellulose column chromatography of HGP-1 and Sephadex G-100 column chromatography of HGP-2 were shown in Figure 3. For the preparation of the elution curve, the collected tube number was on *X*-axis, and the absorbance was on *Y*-axis. In the diagram of DEAE-52 cellulose column chromatography, there were one primary and two secondary peaks of the curve that indicated as protein part, while several minor absorbance peaks were shown as the carbohydrate part (Figure 3A). The coincident absorption peak of the two curves was collected to gain crude glycoprotein HGP-2. In the diagram of Sephadex G-100 column chromatography, there was one primary, and one secondary peak of the curve was shown as the protein part, and one primary and one secondary peak of the curve was indicated as the carbohydrate part (Figure 3B). The coincident absorption peak of the two curves was collected and freeze-dried to gain purified glycoprotein HGP-3.

### 3.6. Analysis of Glycoprotein by SEM, and AFM

**Figure 4:**
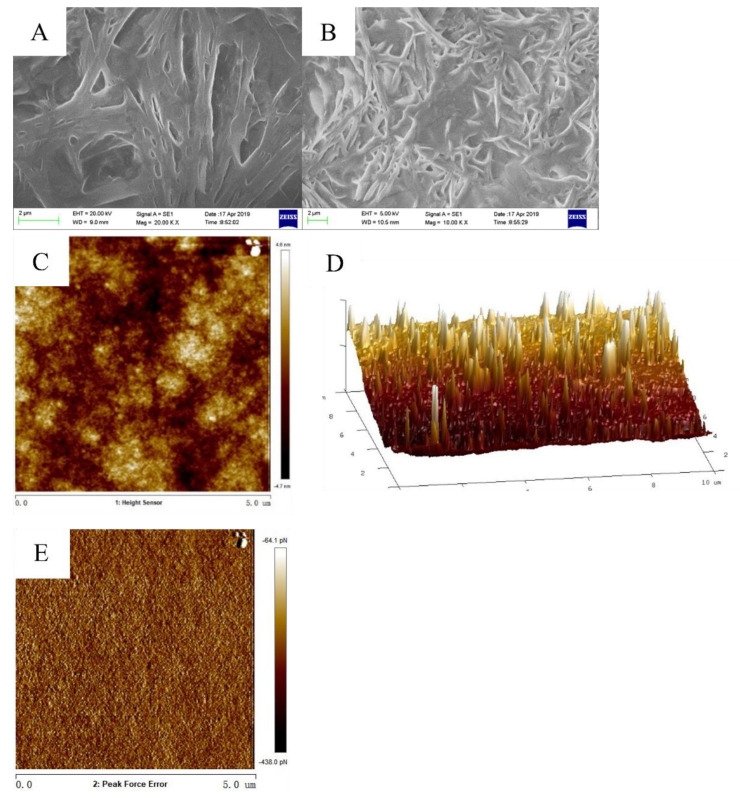
The imaging of scanning electron microscopy of HGP-3 with (**A**) 2000× Mag, (**B**) 1000×. The imaging of atomic force microscopy (AFM) of HGP-3 with (**C**) 2D-images, (**D**) 3D-image, and (**E**) Van Der Waals force image.

As shown in Figure 4A,B, the purified glycoprotein from Hechong (HGP-3) exhibited a similar paralleled fibrous shape containing cracked, shown as scattered among the fibrous protein visualized under Scanning electron microscopy (SEM). HGP-3 formed into fibrous aggregates viewing as threadiness shape visualized under atomic force microscopy (AFM) (Figure 4C,D). The imaging of the Van der Waals force diagram (Figure 4E) indicated that the microspatial structure of purified samples was homogeneous, which was visualized under AFM.

## 4. Discussion

### 4.1. Antioxidant Activities of Extracts

Based on the analyses of DPPH, ABTS, and FRAP, the raw aqueous extracts and steamed Hechong grown in both China and Vietnam have significant antioxidant activities. This is due to the presence of various peptides, polysaccharides, and proteoglycans, which is contributed to antioxidant activities [20]. Many kinds of worms have been proven to have antioxidant capacity. It has been reported that the *Gomphidius rutilus* with worm infection showed higher total phenolic content (TPC) and antioxidant capacity evaluated by ABTS, FRAP, and ORAC (oxygen radical absorbance capacity) than *G. rutilus* without worms [28]. The typical antioxidant assays of DPPH, ABTS, and FRAP are analyzed based on an electron transfer reaction, which involves a redox reaction with the oxidant as an indicator of the reaction endpoint, and as the probe for monitoring the reaction [29].

It is generally recognized that free radicals produced in the body can damage all cellular components, resulting in many chronic diseases such as diabetes, atherosclerosis, cancer, heart failure, and Alzheimer’s disease [30,31]. Thus, determining the free radicals scavenging capability is quite important as the antioxidant activities. All these three methods are similar to each other in the reaction principles; however, slight differences exist based on the reaction time, stability, molecular target, etc., which leads to a similar tendency but different values [32]. For instance, the antioxidant capabilities determined by the assay of ABTS are always higher than the values determined by the DPPH [33]. The possible reasons have been explained that DPPH radicals have relatively higher stability and are relatively harder to be scavenged with longer reaction times [34]. In contrast, when conducting an ABTS assay, time monitoring is most important due to the slight instability of ABTS radicals.

Earlier, the researchers have described that the heating processes such as steaming, boiling, roasting, and frying could affect the nutrient composition in the food, however, these heating processes facilitate the occurrence of higher protein content in steamed fish when compared to that of the fresh fish [35]. Furthermore, the condition of steaming is achieved in a shorter time under boiling water which is similar to that of hot water extraction encompassing prolonged heating. Hence, the outcomes of all three aqueous extracts of steamed Hechong assays presented better antioxidant activities than that of raw Hechong. In the present study, the extracts of 70% acetone have significantly weaker (*p* < 0.05) antioxidant activities than the aqueous extracts do. The solvents with different polarities have an impact on antioxidant activities [30]. Water is the most commonly used solvent for macromolecules extraction of natural resources, while acetone is a solvent with a relatively small polarity that is frequently used in the extraction of small molecules. Relatively low antioxidant capacity presented by 70% acetone extracts of raw Hechong, raw Hechong aqueous extracts, and steamed Hechong aqueous extract showed that the larger molecules contained in Hechong are the main contributors to the antioxidant capability of Hechong. Hence, 70% acetone extraction would decrease the efficiency of performing antioxidant capability. The selected solvent system has important roles for the extracting of distinct antioxidant substances. Changes in the solvent polarity that modifies its capacity to dissolve certain antioxidant compounds and impacts the quantification of antioxidant activity [36].

### 4.2. Antioxidant Activities of Protein Hydrolysates

According to the analyses of DPPH, ABTS, and FRAP, the protein hydrolysates of Hechong exhibited higher antioxidant activities than the raw Hechong having without protease addition. These outcomes coincide with previous studies on the effects of antioxidant activities of protein hydrolysates from bighead carp gill [37] and *Cyprinus carpio* skin gelatin [38]. That is because of various heat treatments, the arrangement of peptide sequences could be changed [39,40,41]. Mostly, the thermal treatment elevates the potential of the hydrolysates and fractions of the peptide to chelate Fe^2+^. Heat processing usually generates biologically active peptides during in vitro enzymatic protein digestion [42], which is associated with several synergistic mechanisms, including the inhibition of free radicals, and the process of lipid peroxidation, countering oxidative reactions by oxygen-containing compounds, and electron transfer [43]. The higher chelating activity of these peptides can be connected to the definite peptide assembly and side-chain groups of amino acids that contribute significant functions either in terminating the free radical chain reactions or in chelating transition-metal ions [44]. Furthermore, amino acids with hydrophobic properties play an important role in providing deter free radicals [45]. Therefore, hydrolysates from Hechong grown in both China and Vietnam have the potential as bioactive hydrolysates with noteworthy antioxidant activity based on the ability to inhibit DPPH· ABTS+· and ferric ion reducing power.

### 4.3. Purification of Glycoprotein from Hechong

Glycoprotein is a biological molecule coupled with oligosaccharides and proteins through covalent bonds, and its functions are very similar to protein. The main functions of glycoproteins are presented in cell recognition, transferring the signals, and transferring the information to protein metabolism [46]. They are normally rich in the extracellular matrix and contribute as a major source of the matrix organization. It is well documented that the oligosaccharide moieties of glycoproteins have an important role in protein stability, function, and turnover [47]. Furthermore, they have pharmacologically potential roles, play as antioxidants, anti-inflammatory, anti-tumor, hypoglycemic, hypolipidemic, anti-aging, and immune-modulatory substances [48].

In this study, the purification of glycoprotein was achieved by DEAE-52 cellulose and Sephadex G-100 gel chromatography columns. The adsorption capacity of activated DEAE-52 cellulose on glycoprotein molecules with different polar groups was found to be different, and the retention capacity of Sephadex G-100 gel on glycoprotein molecules with different molecular weights was also not consistent. Although, relatively high glycoprotein was expected to isolate from Hechong by combining these two kinds of chromatography columns.

The DEAE-52 cellulose with diverse ionic types is very crucial for the separation of glycoproteins. The separation is based on different charges passed on proteins under certain pH and the binding strength of charged groups [49]. Therefore, the charged properties of certain HGP-1 and the binding strength of the charged groups on the cellulose were the same, which could be eluted at the same time however, the separation was not effective using ion-exchange chromatography. Hence, the fraction of the isolated glycoprotein required to be further separated by Sephadex G-100 column chromatography.

Earlier, many researchers have also applied to purify glycoprotein by using DEAE-52 cellulose and Sephadex G-100 column chromatography [46,49,50]. In this study, the column was successively eluted using the buffer solution of 0.1 mol/L NaHCO_3_. There was a single primary and two secondary peaks of the curve observed in the protein part due to the absorbance of 280 nm and 490 nm wavelength, indicating that the fraction consisted of both protein and carbohydrate. Thus, the fraction of the primary peak was collected. After being concentrated and freeze-dried, the yellow protein became a light-yellow glycoprotein, named HGP-2. Further, the elusion of HGP-2 was carried out by deionized water, one primary and one secondary peak of the curve was observed. The fraction in the overlapping absorbance peak was collected at both 280 nm and 490 nm wavelengths. After concentrated and freeze-dried, the white glycoprotein was extracted and purified, named HGP-3.

### 4.4. Purity Identification of Extracted Glycoprotein from Hechong

In this study, the scanning electron microscopy showed the spatial structure of freeze-dried glycoprotein comprising spikes shaped with the homogeneous distribution. HGP exhibited a similar paralleled fibrous shape in the 1000× Mag figure. However, there were some cracked ones scattered among the fibrous protein in the 2000× Mag figure. All the exposed surfaces of glycoprotein showed spike-like surfaces and edges with some serrations, and the width was about 1 μm. Earlier, similar identification of the microarchitecture of glycoprotein matrices obtained from various biological samples have been visualized using scanning electron microscopy [51,52,53].

Atomic force scanning electron microscopy is an effective method to observe the morphology of protein in the airborne environment. It supports detecting the protein indirectly which involves dispersion and aggregation [54]. In this study, the hypsogram and phase diagram of purified glycoprotein showed widespread homogeneous distribution. In both figures, purified glycoproteins formed fibrous aggregates with thread-like shapes.

AFM is a surface-based technology, that requires ligands to be fixed with proteins or their substrate to detect biomolecular interactions. For the AFM measurements, a ligand is attached to a receptor on the surface that enables them to interact [55,56]. AFM detects the displacement of the microcantilever, which depends on the interaction between the sample and the probe. The interaction force is mainly between the last atom at the tip of the needle and the last atom near the surface of the sample [56]. When the distance between the probe and the sample is close to 5 nm, the interaction force between the probe and the sample is the Van der Waals force. In the present study, Van der Waals force ensued in the purified glycoprotein that indicated the microspatial structure of purified samples were homogeneous.

In this study, the outcome of SDS-PAGE, the crude protein HGP-1, and HGP-3 electrophoresis runway mainly consisted of seven bands and a single band respectively. Comparing the outcomes of pre-test and post-test electrophoresis, a glycoprotein from Hechong had been greatly purified since it was observed in a single band. The molecular mass of the glycoprotein was approximately 20 kDa and hence, this small-molecular protein can be readily absorbed in the human body for biological activity. Furthermore, molecular weight and elements measurement accuracy, high-performance liquid chromatography, and mass spectroscopy would be highly recommended.

### 4.5. Limitations and Future Direction

It is easily recognized that some uncontrolled variables existed in this study such as sex, the season of the harvesting, feeding habits, living environment, reproductive status, and so on [35]. All these factors would affect the nutritional compositions and eventually influence the composition of the extracts and protein hydrolysates. Owing to the limitation of sample resources, this study can be established with a larger sampling scale and more groups of data to be analyzed.

With the treatment of hot water extraction and protein hydrolysis, antioxidant activities were significantly higher when compared to the raw Hechong. Based on the discussion in this study, it provides some basic presupposition to develop functional food formula comprising proteins and peptides as it has potent antioxidant properties. The difference between these two is that the former is mainly for extracting glycoprotein peptide from enzymatic hydrolysis, while the latter is to achieve a kind of sports drink that quickly replenishes water, energy, and maintains the basis of the electrolyte balance. Sports drink generally require a variety of glycogen, electrolyte, glycoprotein, and water distribution through the combination of sensory evaluation and the determination of product indicators. Hence, it is a great motive to develop the formula of Hechong sports functional beverages with high quality and taste.

## 5. Conclusions

The antioxidant activities of Hechong could be increased by hot water extraction and protease hydrolysis. Aqueous extracts of raw and steamed Hechong had higher antioxidant activity than other extracts. After protease hydrolysis, the molecular weight distribution of the protein hydrolysate had significantly altered eventually, and the antioxidant activity had also significantly increased. Alcalase, trypsin, and papain hydrolysates with a larger proportion of low-molecule-weight peptides. After hot water extraction, the crude protein was isolated and purified through DEAE-52 cellulose column chromatography and Sephadex G-100 column chromatography to provide purified glycoprotein, HGP-3. In the SDS-PAGE electrophoretogram, there was a single band of HGP-3 found with a molecular mass of around 20 kDa. In the micrographs of SEM and AFM, HGP-3 exhibited a parallel analog of fibrous shapes that aggregated to form a thread-like shape and homogeneous microspatial structure. The glycoprotein from Hechong had purified and tested. Antioxidant activity is generally involved in the treatment of lots of diseases, and glycoprotein might be the most active substance that contributes to those effects. Based on the study, Hechong has great potential values, which is deserved to be further developed and studied in the food and pharmaceutical industries.

## Figures and Tables

**Table 1:** Antioxidant activities of extracts and hydrolysate from Hechong grown in China and Vietnam.

Treatments	China			Vietnam		
	DPPH (μmol TE/g)	ABTS (μmol TE/g)	FRAP (mmol Fe^2+^/100 g)	DPPH (μmol TE/g)	ABTS (μmol TE/g)	FRAP (mmol Fe^2+^/100 g)
Raw Hechong freeze-dried powder	7.38 ± 0.29 ^d^	43.53 ± 0.86 ^d^	1.82 ± 0.10 ^e^	8.83 ± 0.84 ^e^	53.24 ± 2.16 ^d^	2.23 ± 0.21 ^e^
70% Acetone extracts of raw Hechong freeze-dried powder	4.21 ± 0.06 ^e^	11.04 ± 0.59 ^e^	1.18 ± 0.01 ^f^	2.62 ± 0.07 ^f^	6.14 ± 0.24 ^e^	0.64 ± 0.02 ^f^
Raw Hechong aqueous extracts	52.07 ± 2.01 ^b^	114.39 ± 1.84 ^b^	6.77 ± 0.03 ^a^	60.68 ± 2.12 ^b^	135.00 ± 1.48 ^b^	9.93 ± 0.22 ^b^
70% Acetone extracts of raw Hechong aqueous extracts	19.75 ± 0.96 ^c^	54.00 ± 2.00 ^cd^	5.59 ± 0.16 ^c^	19.79 ± 0.26 ^d^	58.25 ± 0.80 ^d^	4.94 ± 0.08 ^d^
Steamed Hechong aqueous extracts	64.15 ± 3.56 ^a^	184.75 ± 15.77 ^a^	6.44 ± 0.07 ^b^	76.29 ± 1.43 ^a^	181.04 ± 6.69 ^a^	10.40 ± 0.36 ^a^
70% Acetone extracts of steamed Hechong aqueous extracts	19.00 ± 0.78 ^c^	61.76 ± 3.42 ^c^	5.42 ± 0.10 ^d^	23.05 ± 0.59 ^c^	71.01 ± 3.89 ^c^	5.40 ± 0.13 ^c^

Data were expressed as Mean ± S.D. (*n* = 3) on a dry weight basis. The DPPH, ABTS, and FRAP values in different groups were marked separately by different letters with significant (*p* < 0.05) differences.

## Data Availability

The datasets generated for this study are available on request to the corresponding author.

## Supplementary Materials

The following supporting information can be downloaded at: https://www.mdpi.com/article/10.3390/foods11131837/s1, Figure S1: Fresh sample of Hechong (A) and the glycoprotein extracts from Hechong at different purified level: crude protein extracts HGP-1 (B) obtained by hot water extraction, crude glycoprotein extracts HGP-2 (C) obtained by DEAE-52 cellulose column chromatography, purified glycoprotein extracts HGP-3 (D) obtained by Sephadex G-100 column chromatography; Table S1: Overview of Hechong extraction methods.

## References

[1] Yang Z., Sunil C., Jayachandran M., Zheng X., Cui K., Su Y., Xu B. (2020). Anti-fatigue effect of aqueous extract of Hechong (*Tylorrhynchus heterochaetus*) via AMPK linked pathway. Food Chem. Toxicol..

[2] Su Y.P., Huang Q., Cui K.P. (2016). Present situation of Hechong industry and analysis of increased breeding benefits in Pearl River Estuary Area. Ocean. Fish..

[3] Wu Y.G., Pang C.F., Wen S.H., Li H.Y. (2006). Analysis and evaluation of nutritional components of Hechong (*Tylorrhynchus heterochaeta*). J. Hydroecology.

[4] Sun H., Yu J., Liang K., An R., You L., Wang X. (2016). Analysis of aliphatic acids in three kinds of nereid and determination of five unsaturated aliphatic acids. Chin. Tradit. Pat. Med..

[5] Jia Y.L., Yan H., Ding G. (2016). Research progress about *Nereis succinea*’s biological activity. J. Zhejiang Ocean. Univ. Nat. Sci..

[6] Deng Z., Wang S., Li Q., Ji X., Zhang L., Hong M. (2010). Purification and characterization of a novel fibrinolytic enzyme from the polychaete, *Neanthes japonica* (Iznka). Bioresour. Technol..

[7] Chen X.H., Yang S., Yang W., Si Y.Y., Xu R.W., Fan B., Wang L., Meng Z.N. (2020). First genetic assessment of brackish water polychaete *Tylorrhynchus heterochaetus*: Mitochondrial COI sequences reveal strong genetic differentiation and population expansion in samples collected from southeast China and north Vietnam. Zool. Res..

[8] Ma D.C., Ye L.H., Xu A.Y., Pan G., Long C. (2014). A histological study of *Tylorrhynchus heterochaetus*. South China Fish. Sci..

[9] Suzuki T., Gotoh T. (1986). The complete amino acid sequence of giant multisubunit hemoglobin from the polychaete *Tylorrhynchus heterochaetus*. J. Biol. Chem..

[10] Green B.N., Suzuki T., Gotoh T., Kuchumov A.R., Vinogradov S.N. (1995). Electrospray ionization mass spectrometric determination of the complete polypeptide chain composition of *Tylorrhynchus heterochaelus* hemoglobin. J. Biol. Chem..

[11] Zheng Y., Ding G., Yang Z., Yu F., Jia Y., Wu Z., Chen R. (2017). Enzymatic preparation and antithrombotic activity of anticoagulant peptides from *Perinereis aibuhitensis* (PAAP). Food Sci..

[12] Liu X., Liu L., Zhao X., Li G., Cai C., Yu G. (2016). Structure characterization and anticoagulant activities of a sulfated polysaccharide from *Perinereis aibuhitensis*. Chin. J. Mar. Drugs.

[13] Zhang R., Feng L., Lei L., Zheng Y., Guo Y. (2014). Separation of the active components from *Nereis Virens* and the mechanism of action against A375 cell. Pharm. Biotechnol..

[14] Day L., Seymour R.B., Pitts K.F., Konczak I., Lundin L. (2009). Incorporation of functional ingredients into foods. Trends Food Sci. Technol..

[15] Halim N.R.A., Azlan A., Yusof H.M., Sarbon N.M. (2018). Antioxidant and anticancer activities of enzymatic eel (*Monopterus* sp.) protein hydrolysate as influenced by different molecular weight. Biocatal. Agric. Biotechnol..

[16] Firmansyah M., Abduh M.Y. (2019). Production of protein hydrolysate containing antioxidant activity from *Hemetia illucens*. Heliyon.

[17] Putra S.N.K.M., Ishak N.H., Sarbon N.M. (2018). Preparation and characterization of physicochemical properties of golden apple anail (*Pomacea canaliculata*) protein hydrolysate as affected by different proteases. Biocatal. Agric. Biotechnol..

[18] Oliveira S.R.M., Nascimento A.E., Lima M.E., Leite Y.F., Benevides N.M. (2002). Purification and characterisation of a lectin from the red marine alga *Pterocladiella capillacea* (S.G. Gmel.) Santel. & Hommers. Braz. J. Bot..

[19] Guo H., Deng W.X., Zhang Y. (2009). Research progression of glycoprotein. Biotechnol. Bull..

[20] Chen J., Jayachandran M., Xu B., Yu Z. (2019). Sea bass (*Lateolabrax maculatus*) accelerates wound healing: A transition from inflammation to proliferation. J. Ethnopharmacol..

[21] Altinelataman C., Koroleva O., Fedorova T., Torkova A., Lisitskaya K., Tsentalovich M., Kononikhin A., Popov I., Vasina D., Kovalyov L. (2019). An in vitro and in silico study on the antioxidant and cell culture-based study on the chemoprotective activities of fish muscle protein hydrolysates obtained from European seabass and gilthead seabream. Food Chem..

[22] Tan X., Qi L., Fan F., Guo Z., Wang Z., Song W., Du M. (2018). Analysis of volatile compounds and nutritional properties of enzymatic hydrolysate of protein from cod bone. Food Chem..

[23] Kirk P.L. (1950). Kjedahl method for total nitrogen. Anal. Chem..

[24] Luo J., Cai W., Wu T., Xu B. (2016). Phytochemical distribution in hull and cotyledon of adzuki bean (*Vigna angularis* L.) and mung bean (Vigna radiate L.), and their contribution to antioxidant, anti-inflammatory and anti-diabetic activities. Food Chem..

[25] Ismail A., Azlan A., Khoo H.E., Prasad K.N., Kong K.W. (2013). Antioxidant Assays: Principles, Methods and Analysis.

[26] Chen J., Jayachandran M., Zhang W., Chen L., Du B., Yu Z., Xu B. (2019). Dietary supplementation with Sea Bass (*Lateolabrax maculatus*) ameliorates ulcerative colitis and inflammation in macrophages through inhibiting Toll-like receptor 4-linked pathways. Int. J. Mol. Sci..

[27] Beeley J.G. (1986). Glocoprotein and Proteoglycan Techniques.

[28] Sun L., He W., Xin G., Cai P., Zhang Y., Zhang Z., Wei Y., Sun B., Wen X. (2018). Volatile components, total phenolic compounds, and antioxidant capacities of worm-infected *Gomphidius rutilus*. Food Sci. Hum. Wellness.

[29] Huang D., Ou B., Prior R.L. (2005). The chemistry behind antioxidant capacity assays. J. Agric. Food Chem..

[30] Xu B.J., Chang S.K.C. (2007). A comparative study on phenolic profiles and antioxidant activities of legumes as affected by extraction solvents. J. Food Sci..

[31] Ganesan K., Xu B. (2017). A critical review on polyphenols and health benefits of black soybeans. Nutrients.

[32] Letha N., Ganesan K., Nair S.K.P., Gani S.B. (2016). Studies on phytochemical screening and in vitro antioxidant activity of Ethiopian indigenous medicinal plants, *Artemisia abyssinica* Sch. Bip. Ex A.Rich. World J. Pharma. Res..

[33] Xu B., Ganesan K., Mickymaray S., Alfaiz F.A., Thatchinamoorthi R., Al Aboody M.S. (2020). Immunomodulatory and antineoplastic efficacy of common spices and their connection with phenolic antioxidants. Bioact. Compd. Health Dis..

[34] Tokusoglu O., Hall C. (2011). Fruit and Cereal Bioactives (Sources, Chemistry, and Applications).

[35] Puwastien P., Judprasong K., Kettwan E., Vasanachitt K., Nakngamanong Y., Bhattacharjee L. (1999). Proximate composition of raw and cooked Thai freshwater and marine fish. J. Food Compos. Anal..

[36] Zhou K., Yu L. (2004). Effects of extraction solvent on wheat bran antioxidant activity estimation. LWT Food Sci. Technol..

[37] Lin J., Hong H., Zhang L., Zhang C., Luo Y. (2019). Antioxidant and cryoprotective effects of hydrolysate from gill protein of bighead carp (*Hypophthalmichthys nobilis*) in preventing denaturation of frozen surimi. Food Chem..

[38] Tkaczewska J., Borawska-Dziadkiewicz J., Kulawik P., Duda I., Morawska M., Mickowska B. (2019). The effects of hydrolysis condition on the antioxidant activity of protein hydrolysat from *Cyprinus carpio* skin gelatin. LWT Food Sci. Technol..

[39] Zielińska E., Baraniak B., Karaś M. (2017). Antioxidant and anti-Inflammatory activities of hydrolysates and peptide fractions obtained by enzymatic hydrolysis of selected heat-treated edible insects. Nutrients.

[40] Matheswaran P., Raja L., Gani S.B. (2019). Antioxidant and anti-inflammatory efficacy of functional proteins obtained from seven edible insects. Int. J. Entomol. Res..

[41] Matheswaran P., Raja L., Gani S.B. (2020). Anti-Hypertensive and Anti-microbial activity of protein hydrolysate obtained from seven edible insects. Bull. Pure Appl. Sci..

[42] Karaś M., Baraniak B., Rybczyńska-Tkaczyk K., Gmiński J., Gaweł-Bęben K., Jakubczyk A. (2015). The influence of heat treatment of chickpea seeds on antioxidant and fibroblast growth-stimulating activity of peptide fractions obtained from proteins digested under simulated gastrointestinal conditions. Int. J. Food Sci. Technol..

[43] Chen H., Muramoto K., Yamauchi F., Nokihara K. (1996). Antioxidant activity of designed peptides based on the antioxidative peptide isolated from digests of a soybean protein. J. Agric. Food Chem..

[44] Zhu L., Chen J., Tang X., Xiong Y.L. (2008). Reducing, radical scavenging, and chelation properties of in vitro digests of alcalase-treated zein hydrolysate. J. Agric. Food Chem..

[45] Saadi S., Saari N., Anwar F., Hamid A.A., Ghazali H.M. (2015). Recent advances in food biopeptides: Production, biological functionalities therapeutic applications. Biotechnol. Adv..

[46] Yang J.-T., Wu C.-E., Li Y.-Y., Jia S.-Q., Fan G.-J., Peng F.-R. (2011). Identification and purification of an allergic glycoprotein from *Ginkgo biloba* Kernel. Agric. Sci. China.

[47] Kumar G., Murugesan A.G. (2007). Influence of *Helicteres isora* bark extracts on plasma and tissue glycoprotein components in streptozotocin diabetic rats. J. Clin. Diagn. Res..

[48] Zeng H.-j., Liu Z., Wang Y.-P., Yang D., Yang R., Qu L.-B. (2018). Studies on the anti-aging activity of a glycoprotein isolated from Fupenzi (*Rubus chingii* Hu.) and its regulation on klotho gene expression in mice kidney. Int. J. Biol. Macromol..

[49] Zheng W., Zhao T., Feng W., Wang W., Zou Y., Zheng D., Takase M., Li Q., Wu H., Yang L. (2014). Purification, characterization and immunomodulating activity of a polysaccharide from flowers of *Abelmoschus esculentus*. Carbohydr. Polym..

[50] Sun Y.J., Chen Y., Song Z.-Y., Zhou D.-W. (2007). Purification and analysis of *Cimicifuga foetida* glycoprotein (CF-I). Zhong Yao Cai.

[51] Familiari G., Heyn R., Petruzziello L., Relucenti M., Schatten H. (2012). A Method to Visualize the Microarchitecture of Glycoprotein Matrices with Scanning Electron Microscopy, in Scanning Electron Microscopy for the Life Sciences.

[52] Bjugn R., Flood P.R. (1988). Scanning electron microscopy of human urine and purified Tamm-Horsfall’s glycoprotein. Scand. J. Urol. Nephrol..

[53] Mistri A., Kumari U., Mittal S., Mittal A.K. (2020). Modifications in the gills of hill stream Moth catfish, *Hara hara* (Erethistidae, Siluriformes): A light and scanning electron microscope investigation. Tissue Cell.

[54] Akkermans C., Van der Goot A.J., Venema P., Gruppen H., Vereijken J.M., Van der Linden E., Boom R.M. (2007). Micrometer-sized fibrillar protein aggregates from soy glycinin and soy protein isolate. J. Agric. Food Chem..

[55] Le D.T.L., Guerardel Y., Loubière P., Mercier-Bonin M., Dague E. (2011). Measuring kinetic dissociation/association constants between. Biophys. J..

[56] Klinov D.V., Protopopova A.D., Andrianov D.S., Litvinov R.I., Weisel J.W. (2020). An improved substrate for superior imaging of individual biomacromolecules with atomic force microscopy. Colloids Surf. B Biointerfaces.

