# Supplementary material for: Antioxidant Activities of Aqueous Extracts and Protein Hydrolysates from Marine Worm Hechong (Tylorrhynchus heterochaeta)
Source: Foods. 2022 Jun 22;11(13):1837. doi: 10.3390/foods11131837 (PMC9265576; doi:10.3390/foods11131837)
Supplement: Supplementary file 1 [file foods-11-01837-s001.zip › foods-1759490-Supplemental Table and figure.pdf]

foods

MDPI

Supplementary Table S1. Overview of Hechong extraction method.

Group No.	The Method of Extraction and Briefing the Protocol
1	Directly freeze-drying raw Hechong to obtain a freeze-dried powder.
2	Extracted raw Hechong freeze-dried powder using 70% acetone.
3	Extracted raw Hechong by hot water extraction, and freeze-drying the supernatant.
4	Extracted raw Hechong aqueous extracts using 70% acetone.
5	Extracted steamed Hechong by hot water extraction, and freeze-drying the supernatant
6	Extracted steamed Hechong aqueous extracts using 70% acetone

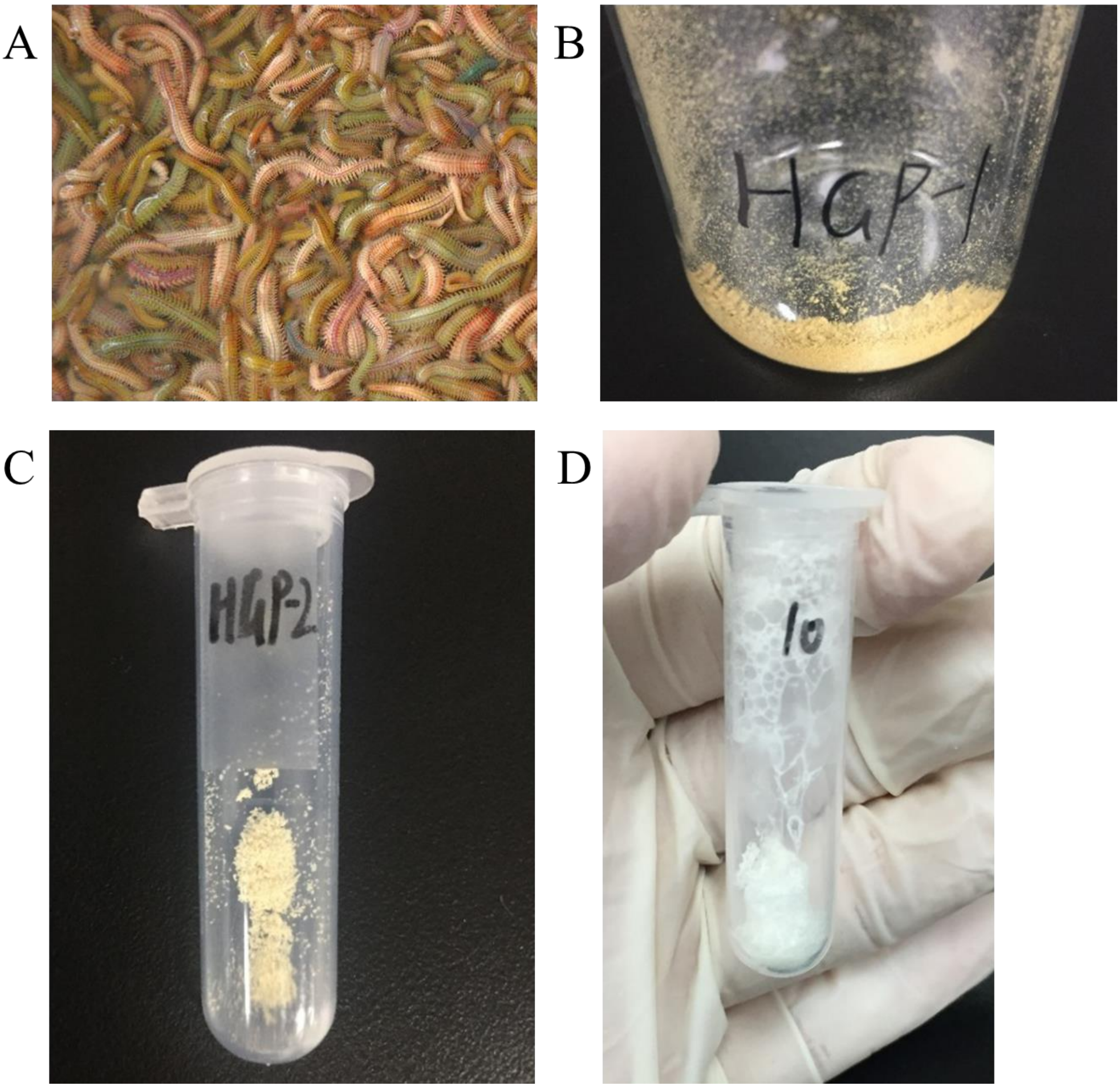

Supplementary Figure S1. (**A**) Fresh sample of Hechong. (**B**) Crude protein extracts of HGP-1 by hot water extraction (**C**) crude glycoprotein extracts of HGP-2 by DEAE-52 cellulose column chromatography, (**D**) purified glycoprotein extracts of HGP-3 obtained by Sephadex G-100 column chromatography.
